# Comparison of three different bioleaching systems for Li recovery from lepidolite

**DOI:** 10.1038/s41598-020-71596-5

**Published:** 2020-09-03

**Authors:** J. Sedlakova-Kadukova, R. Marcincakova, A. Luptakova, M. Vojtko, M. Fujda, P. Pristas

**Affiliations:** 1 Faculty of Science, Pavol Jozef Safarik University in Kosice, Srobarova 2, 04154 Kosice, Slovakia; 2 Faculty of Material, Metallurgy and Recycling, Technical University of Kosice, Letna 9, 04200 Kosice, Slovakia; 3 Institute of Geotechnics, Slovak Academy of Sciences, Watsonova 45, 04001 Kosice, Slovakia; 4 Institute of Materials Research, Slovak Academy of Sciences, Watsonova 47, 04001 Kosice, Slovakia

**Keywords:** Metals, Biotechnology, Microbiology, Biogeochemistry, Environmental sciences

## Abstract

Three different biological systems, the consortium of autotrophic bacteria *Acidithiobacillus ferrooxidans* and *Acidithiobacillus thiooxidans*, heterotrophic fungus *Aspergillus niger* and heterotrophic yeast *Rhodotorula mucilaginosa*, were investigated for lithium extraction from lepidolite. The bacterial consortium was the most effective, 11 mg l^−1^ of Li was dissolved in the absence of nutrients within 336 days. Fungal and yeast bioleaching was faster (40 days), however, with lower extraction efficiency. Bioaccumulation represented a main process of Li extraction by *R. mucilaginosa* and *A. niger*, with 92 and 77% of total extracted Li accumulated in the biomass, respectively. The X-ray diffraction analysis for bioleaching residue indicated changes caused by microorganisms, however, with differences between bacterial leaching and bioleaching by fungi or yeasts. The final bioleaching yields for bacterial consortium, *A. niger* and *R. mucilaginosa* were 8.8%, 0.2% and 1.1%, respectively. Two-step bioleaching using heterotrophic organisms followed by autotrophic bioleaching could lead to the increase of the process kinetics and efficiency. Bioaccumulation of Li offers strong advantage in Li extraction from solution.

## Introduction

Ranking as the lightest alkaline metal, lithium is widely used in metallurgy, aerospace, ceramic, battery and fuel cell industries especially owing to its unique electrochemical reactivity and other properties as well^1^. The increased usage in lithium ion batteries to power portable consumer electronics and electric vehicles results in rising demand for lithium. According to several market research companies huge increase in lithium production is predicted, counting for 66% increase of global lithium production by 2025^2^. Over the period 2021 to 2023 a rapid deficiency of Li may be expected^3^. Therefore, in the coming years lithium demand will rapidly increase.

In nature, lithium is present in lake brines, pegmatites and sedimentary rocks^4^. More than 80% of today’s lithium is obtained from brines^5^. Since the lithium demand has significantly increased in the past years the lithium-containing ores have regained a great importance^6^. Therefore, developing the technology of extracting lithium from solid lithium ores will be important to meet the demand for lithium. Compared to brines the extraction of lithium from hard rock is much more difficult and involves a number of extra operations such as beneficiation to give a concentrate containing 1–3% Li and also roasting in sulphate or carbonate to receive Li into water-soluble species^7^.

One of the main industrial minerals of lithium is spodumene because it has the largest deposits over the world and it does not contain many other metals. However, the most abundant Li ore is lepidolite, a type of pegmatite, that has an ideal formula of K(Li,Al)_3_(SiAl)_4_O_10_(F,OH)_2_, and its distribution is much wider than that of lithium brine. The content of Li_2_O in lepidolite is relatively low ranging in 3.0–7.7 wt.% (containing 1.39–3.58% of Li) comparing with that of spodumene (6–8 wt.%) ^8^. The lithium extraction from lepidolite often incurs higher costs owing to low utilization of other metals contained in the lepidolite^9^. Numerous new procedures (sulfuric acid, lime, sulfate, etc.) were studied to be used for lithium recovery from lepidolite^10,11^. However, the use of lepidolite in hydrometallurgy is restricted by high cost of the hydrometallurgical process for lithium recovery from this mineral as it requires a high concentration of acid and complex purification processes^10^. Alternative technology represents utilisation of bioleaching which becomes viable owing to reduced costs, higher efficiency and green processing^12^. Owing to its special properties metal bioleaching has gradually replaced the hydrometallurgical methods.

Despite the various advantages of bioleaching its application on Li recovery from hard rock ores is scarce. Up to now just studies of the Rezza et al.^13,14^ and Reichel et al.^15^ were published. Rezza et al.^13,14^ reported the utilisation of heterotrophic microscopic fungi *Penicillium purpurogenum, Aspergillus niger* and yeast *Rhodotorula rubra* for spodumene bioleaching. They recovered 1.26, 0.75 and 1.53 mg l^−1^ of Li in nutrient rich medium and 1.06, 0.37 and 0.5 mg l^−1^ of Li in nutrient poor medium by *P. purpurogenum, A. niger* and yeast *R. rubra*, respectively. Reichel et al.^15^ for the first time reported the application of autotrophic bacteria for zinnwaldite bioleaching. They used un-identified, adapted mixed culture of sulphur-oxidising bacteria obtained from leaching of sulphide tailings. They reported 11% recovery of Li in batch experiments with sulphur addition and 26% Li recovery in bioreactor experiments. The first application of bioleaching to lepidolite was recorded by our group^16–18^, however, only few factors influencing the process were investigated. To conduct a comprehensive study of lithium bioextraction from lepidolite we focused on three different microbial systems, the consortium of autotrophic bacterial strains of *Acidithiobacillus ferrooxidans* and *Acidithiobacillus thiooxidans*, heterotrophic fungus *Aspergillus niger* and heterotrophic yeast *Rhodotorula mucilaginosa.* The selected microorganisms are widespread in nature and participate in bioweathering of rocks, mobilization of metals from minerals, in metal precipitation and deposition and are widely applied in biohydrometallurgical processes. Our aim was to study and compare the kinetics of bioleaching by the three biological systems, changes in mineral structures and contribute to the understanding of mechanisms responsible for Li bioextraction from hard rock ores.

## Materials and methods

### Materials

The crushed lepidolite used in this work was provided by prof. Rowson (University of Birmingham, UK). It was ground in ball mill and sieved to less than 150 μm with approximately 75% of particles bellow 100 μm. The mineralogical deposit of the ore is situated in Beauvoir (France). The composition of this mineral is shown in Table 1. Content of the lithium in lepidolite was determined by AAS as 1.21%.

All chemicals used in the experiments were analytical grade reagents. Deionised water was used to prepare solutions for the experimental procedures and also for the analytical tests.

### Microorganisms

The fresh culture of *Aspergillus niger* strain An-S (isolated from the acidified site of Šobov near Banská Štiavnica with a high content of exchangeable aluminium) was obtained from Department of Soil Science, Faculty of Natural Sciences in Bratislava and maintained at 4 °C on a solid Sabourad Dextrose Agar (HiMedia Laboratories) slant. The fungal strain is registered in the Collection of Microscopic Fungi ISB in České Budejovice under the number 1674^19^ Stock cultures were subcultured every month.

Pure cultures of *Acidithiobacillus ferrooxidans* strain SmolnikLC and *A. thiooxidans* strain SmolnikF were obtained from Institute of Geotechnics, Slovak Academy of Sciences in Košice and maintained at 4 °C in 9 K and Waksman and Joffe media, respectively. Both bacteria were isolated from copper mine drainage in Smolník region, Slovakia.

Pure culture of *Rhodotorula mucilaginosa* CCY 20-1-36 (former name *Rhodotorula rubra*) was obtained from Collection of Yeast Cultures from Chemical Institute of Slovak Academy of Sciences in Bratislava.

### Bioleaching experiments

#### Bioleaching by *A. niger*

According to our previous study^16^ biomass received after 8-days spores’ cultivation was used in experiment as the age of spores or conidia of the heterotrophic fungus influenced lithium dissolution from the mineral and a higher Li bioleaching efficiency was achieved using long-term cultured fungi.

The experiments were carried out in 250 ml Erlenmeyer flasks containing 200 ml of standard liquid bioleaching media composed of glucose—5 g l^−1^ and (NH_4_)_2_SO_4_—0.5 g l^−1^ with the initial pH value of 5.1 adjusted using 10 M H_2_SO_4_. To each bioleaching media 2 g crushed mineral and 5 ml of 8-day old conidia (biomass), were added. The flasks were sealed with removable cotton and the experiment was carried out at 21 °C statically. Prior to leaching the medium and mineral were sterilized by autoclaving for 20 min at 120 °C. At pre-determined intervals (4, 11, 18, 25, 33 and 41 day) 5 ml of media were collected by disposable sterile pipettes and filtered through the 0.45 µm-pore-size membrane filter. At the end of the experiments the biomass was easily removed by tweezers and washed with deionised water. The bioleaching residue was obtained after the filtering the rest of the medium and washed with deionised water. The biomass and residue samples were air-dried for 24 h and consequently mineralised in oven for 4 h at 500 °C. Thereafter, the biomass was digested by the 2 M HCl to determine lithium accumulated in the biomass. The amount of biomass was calculated per 1 l of media. To calculate Li recovery efficiency Li concentration was analysed in filtrate, in the biomass and bioleaching residue. Each treatment was prepared in duplicate (two flasks for each pre-determined withdrawal time were prepared). Control experiments with the same media just without microorganisms addition were carried out simultaneously.

#### Bioleaching by consortium of *A. ferrooxidans* and *A. thiooxidans*

The experiments were conducted in 250 ml Erlenmeyer flasks containing 190 ml of nutrient rich or poor medium. Composition of nutrient medium for bacterial consortium was adapted from basic media for individual acidithiobacilli and consist of KH_2_PO_4_—0.1 g, (NH_4_)_2_SO_4_—2 g, KCl—0.1 g, MgSO_4_.7H_2_O—4 g, FeSO_4_.7H_2_O—44.2 g and elemental sulphur 4 g in 1,000 ml of deionised water. The poor medium was prepared just as a solution of H_2_SO_4_ with pH 3 and elemental sulphur 5 g l^−1^. Medium pH prior bacteria addition was adjusted to 3 by 10 M H_2_SO_4_. 10 ml of adapted bacterial consortium was added. After pH dropped to 1.5 due to production of sulphuric acid, crushed lepidolite was added in concentration 10 g l^−1^. Control experiments with the same medium but without microorganisms were set up. All culture and control flasks were incubated at 30 °C, statically. All experiments were carried out in duplicate. At regular intervals (day 1, 3, 7, 10, 14, 21, 49, 77, 171, 205 and 366) samples were withdrawn using 0.2 µm-pore-size membrane filters and supernatant was analysed for Li content using AAS. At the end of the experiments the bioleaching residue was obtained after the filtering the rest of the medium and washed with deionised water. The residue samples were air-dried for 24 h and analysed for Li content by AAS.

Adaptation of bacteria to lepidolite was carried out prior bioleaching experiments and it lasted for two months. Bacteria were cultivated in 180 ml of nutrient medium with addition of 10 ml of pure culture of *A. ferrooxidans* and 10 ml of *A. thiooxidans* pure culture and 10 g l^−1^ of lepidolite.

#### Bioleaching by *R. mucilaginosa*

The experiments were carried out in 250 ml Erlenmeyer flasks containing 200 ml of bioleaching media, yeast cells pre-cultivated 5 days in Petri dish prior the experiments and 10 g l^−1^ of crushed ore. Two types of media, rich and poor, were used for experiments. Composition of rich medium was in 1,000 ml deionised water—glucose—20 g, KH_2_PO_4_—5 g, (NH_4_)_2_SO_4_—5 g, MgSO_4_—0.34 g and yeast extract 7 g. Poor medium consist of glucose—5 g and (NH_4_)_2_SO_4_—0.5 g in 1,000 ml of deionised water. The medium pH was adjusted to 5.1 by 10 M H_2_SO_4_ and sterilized by autoclaving for 20 min at 120 °C before biomass was added. Flasks were placed in shaker at 160 rpm. At pre-defined intervals (days 6, 13, 20, 27, 34, 42, 52 and 59) the samples were collected using micropipettes and centrifuged at 4,500 g for 5 min. Li concentration was analysed in supernatant by AAS. Experiments were carried out at 21 °C, statically. All were conducted in duplicate. Control bioleaching was conducted at the same way without yeast addition.

At the end of the experiments, remaining supernatant with cells was collected by pipette and centrifuged at 4,500 g for 10 min to obtain a biomass. The bioleaching residue was obtained after the filtering the rest of the medium and washed with deionised water. The biomass and residue samples were air-dried for 24 h and consequently mineralised in oven for 4 h at 500 °C. Thereafter, the biomass was digested by the 2 M HCl to determine lithium accumulated in the biomass. The amount of biomass was calculated per 1 l of media. To calculate Li recovery efficiency Li concentration was analysed in filtrate, in the biomass and bioleaching residue.

### Analytical and characterisation methods

Solution pH was measured using a GRYF 208L pH meter with a combined electrode. Li concentration in aqueous samples was measured by Atomic Absorption Spectrophotometer (Perkin Elmer 3,100) at 670 nm. The initial sample and final leaching residues were also mounted with silver paste on aluminium stubs, then coated with 300 – 400 A Au/Pd in a sputtering unit and finally examined in a JEOL scanning electron microscope (JEOL JSM-35CE). Mineral composition before and after the bioleaching process was determined by a diffractometer Bruker D2 Phaser (Bruker AXS, GmbH, Germany) in Bragg–Brentano geometry (configuration Theta-2Theta), CuKα radiation.

## Results and discussion

### Bioleaching kinetics

**Figure 1:**
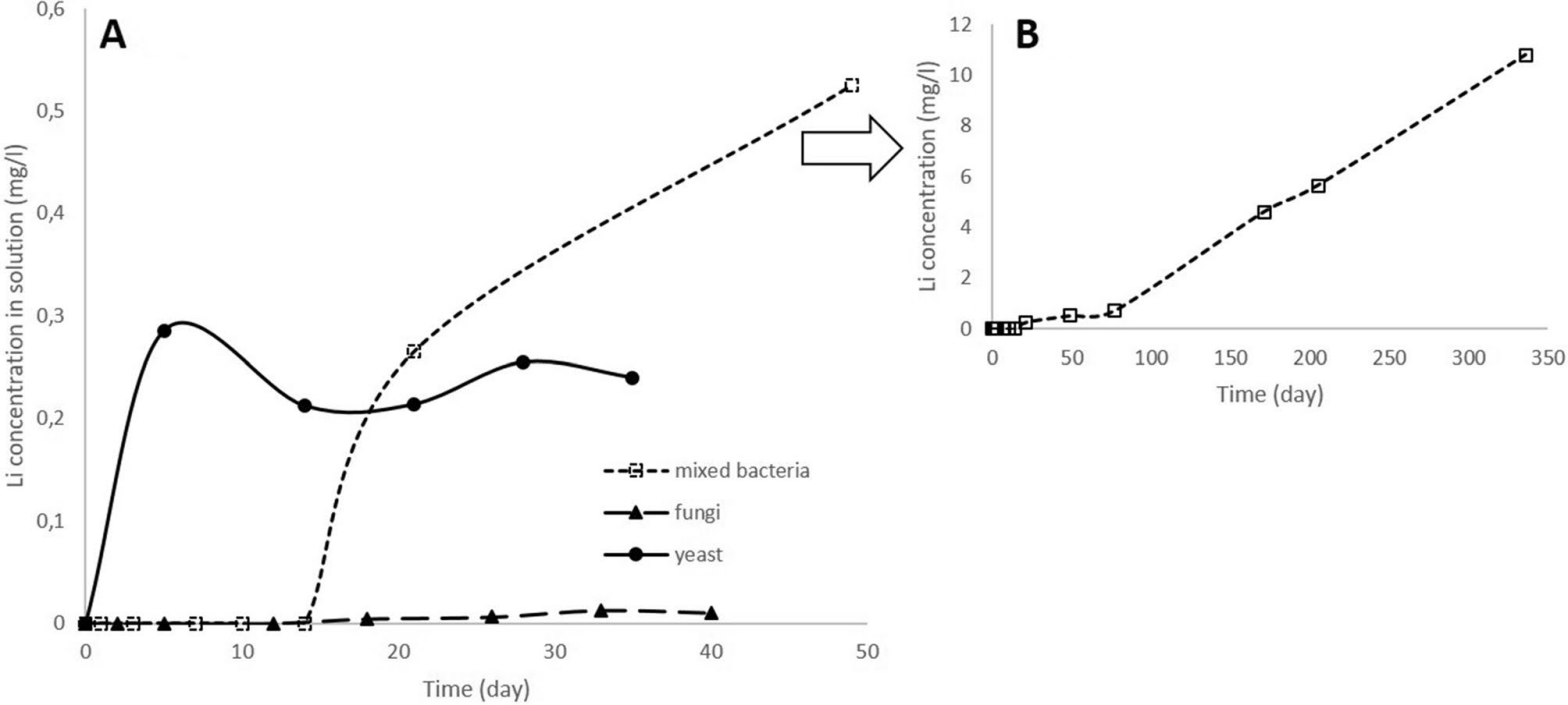
Kinetics of Li bioleaching from lepidolite by consortium of *A. ferrooxidans* and *A. thiooxidans* (bacteria), *A. niger* (fungi) and *R. mucilaginosa* (yeast) (**A**), long-term kinetics of Li bioleaching by bacteria (**B**) (fungi: initial ore concentration 10 g l^−1^, t = 21 °C, pH = 5.1, statically, standard medium, yeast: 10 g l^−1^, t = 21 °C, pH = 5.1, shaking 160 rpm, rich medium and bacteria: 10 g l^−1^, t = 30 °C, pH = 1.5, statically, poor medium).

Comparison of Li bioleaching by three various types of organisms (Fig. 1) revealed that the leaching kinetics in systems with yeast *R. mucilaginosa* was the fastest. Presence of Li in solution was detected at 6th day of the process. After initial faster bioleaching within first 6 days (285.5 µg l^−1^), there was a gradual decrease of Li concentration in solution due to Li bioaccumulation into the biomass up to 13th day and later stable Li concentration in range of 240–250 µg l^−1^ was observed suggesting that the rate of bioleaching and bioaccumulation were equal.

The lowest amount of Li was bioleached by fungi *A. niger*. Under this bioleaching conditions Li was for the first time observed in solution after 26 days of the process. Its concentration gradually increased later on. Again bioaccumulation was observed affecting the amount of Li in the solution.

In the case of bacteria, medium composition was the most important for Li bioleaching. In nutrient rich medium for acidophilic chemoautotrophic acidithiobacilli which contained energy sources (Fe^2+^ ions and S^0^) no Li bioleaching was observed during the whole process time. However, in the medium with limited amount of nutrients and energy sources containing just sulphuric acid and elemental sulphur, Li^+^ ions presence was observed at 21st day for the first time. Bacteria were probably forced to utilize nutrients necessary for their life directly in the leached material. During the first 77 days the lithium bioleaching kinetics was very slow but this stage was followed by the sharp increase of bioleaching rate (400 times increase of the bioleaching rate was observed) resulting in 11 mg l^−1^ of solubilised Li at the end of the bioleaching experiments (after 336 days). The rapid change in the bioleaching rate might be attributed to the changes of mineral structure due to bacterial activity. No Li was found in control experiments using the media without microorganisms addition.

#### Kinetic analysis

To kinetically interpret the heterogeneous non-catalytic reaction for lepidolite bioleaching the shrinking core model (SCM) was used. The assumptions to use the model are based on the three facts—(i) mixed lepidolite particles are considered as nonporous particles, (ii) ore grains gradually shrank and (iii) the product layers form around the unreacted grains^20^. The development and verification of the model were previously described in details by several authors^20,21^.

**Figure 2:**
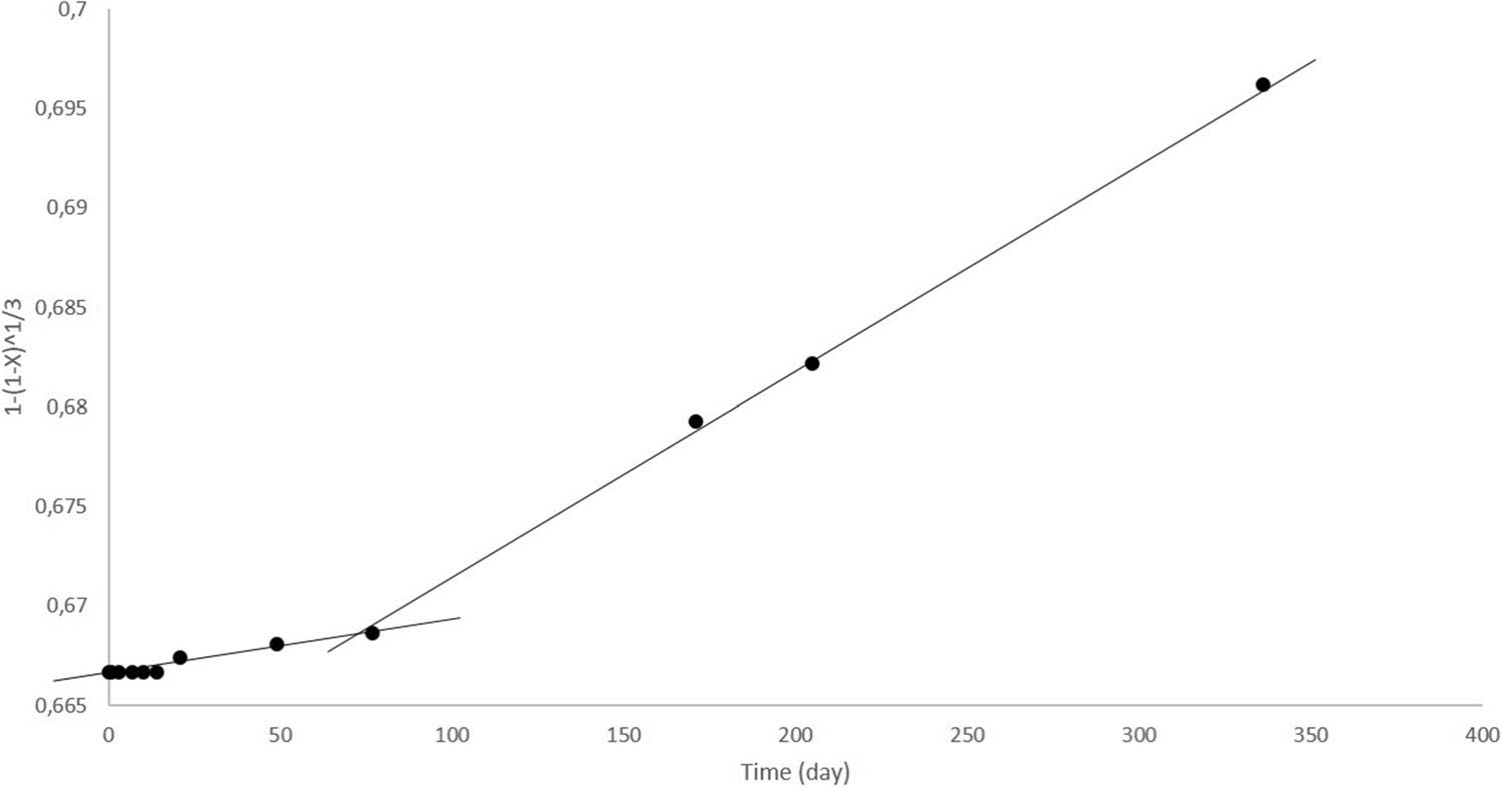
Plot of 1−(1−X)^1/3^ versus time for Li recovery by consortium of bacteria (initial ore concentration 10 g l^−1^, t = 30 °C, pH = 1.5, statically, poor media).

Experimental data obtained for all three studied bioleaching systems were substituted into both equations of SCM model. In the case of bacterial bioleaching a plot of 1−(1−X)^1/3^ versus time (Fig. 2) was found a straight line suggesting that chemical reaction and outer diffusion are the rate controlling steps of the process of bacterial bioleaching. Changes of rate constant, k_r_, (apparent from slopes of the plots) can be visible, as well. The linear relationship was obtained in the initial stage of bioleaching (R^2^ = 0.9944) and later at the day 77 the rate of the process changed but still showed the good fitting obtained by plotting 1−(1−X)^1/3^ versus time (R^2^ = 0.9991). This changes are very well visible also in the previous Fig. 1 showing the increase of Li^+^ ion concentration within the experimental period.

However, the SCM model did not fit to the bioleaching data of two other bioleaching systems, using fungi and yeasts. Obviously, parallel bioaccumulation of Li^+^ ions into the biomass was responsible for considerably different bioleaching behaviour.

### Changes of pH

**Figure 3:**
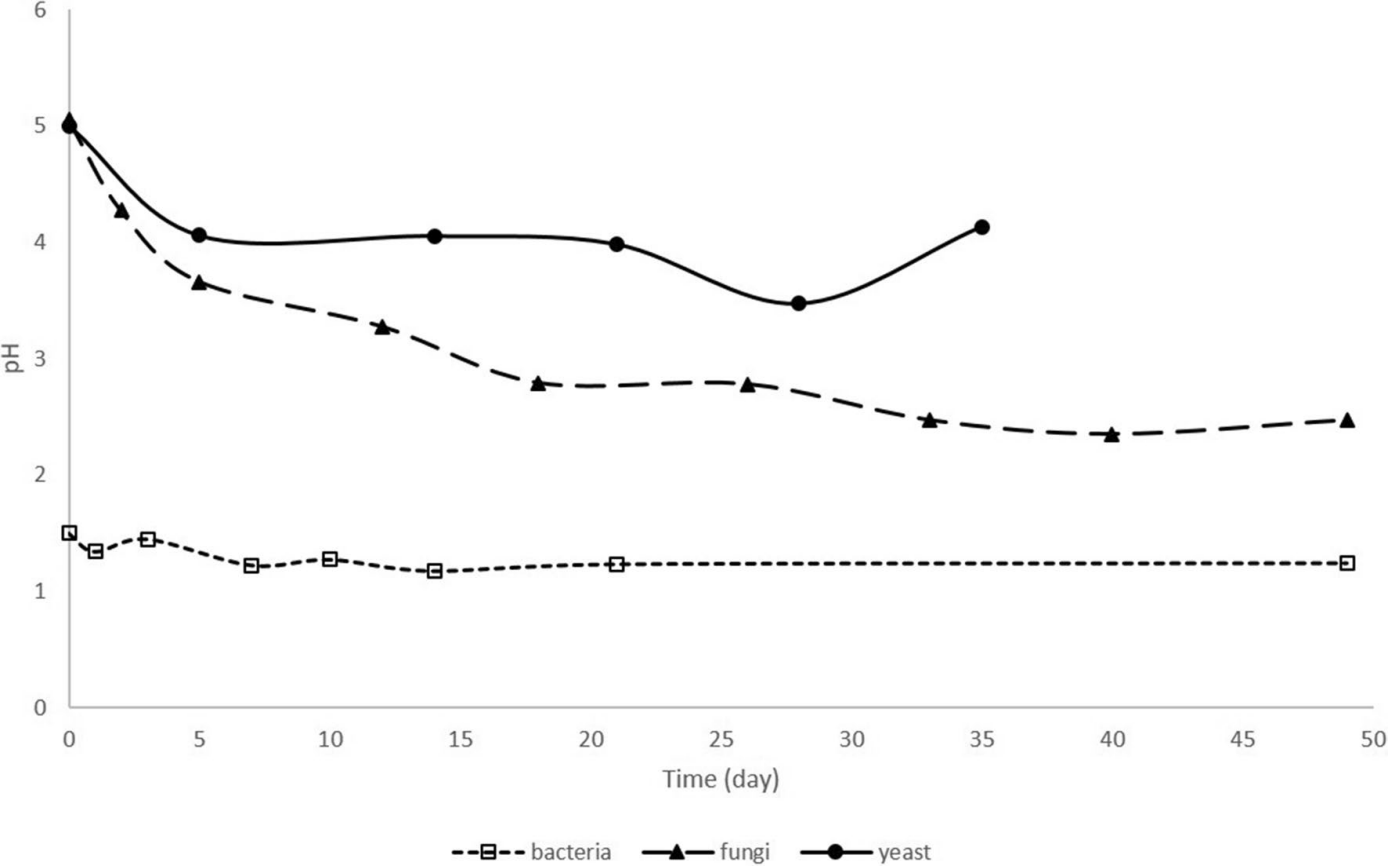
Changes of pH during bioleaching of lepidolite by consortium of *A. ferrooxidans* and *A. thiooxidans* (bacteria), *A. niger* (fungi) and *R. mucilaginosa* (yeast) (fungi: initial ore concentration 10 g l^−1^, t = 21 °C, pH = 5.1, statically, standard medium, yeast: 10 g l^−1^, t = 21 °C, pH = 5.1, shaking 160 rpm, rich medium and bacteria: 10 g l^−1^, t = 30 °C, pH = 1.5, statically, poor medium).

Conditions of bioleaching experiments (pH, medium composition) were adjusted according the type of the microorganism used. Independently of conditions, the decrease of pH (Fig. 3) was recorded in all three bioleaching system. The most obvious decrease in pH occurred in bioleaching by microscopic fungi *A. niger*, with a pH decrease from 5.1 to 3 within first 12 days, followed by slow decrease to 2.5 until the end of the experiment. According to various authors^22,23^, it can be suggested that organic acids, considered the main fungal bioleaching agents, were produced. In the control medium a small increase in pH (from 5.2 to 5.6) was observed.

A similar pattern was also observed in bacterial bioleaching, in which fast decrease of pH to 1.2 was observed during first 7 days followed by slow decrease to 0.9. Later the pH was stable in range of 0.9–1.2. Probably bacteria *A. thiooxidans* were mainly responsible for such pH decrease. In the control without bacteria addition the pH initially decreased from 1.5 to 1.3 and later increased and remained at 1.5.

As shown in Fig. 3 fast pH decrease was observed during first 6 days of bioleaching with yeast *R. mucilaginosa* from initial 5.1 to 4.1. Later pH did not change until 20th day followed by slow decrease to 3.5 at 30th day. In control media, without microorganisms, pH value slowly increased from initial 5.1 to final 5.5.

### Bioleaching mechanisms

According to obtained results different mechanisms can be suggested for lepidolite bioleaching by biological systems studied. Mechanisms of Li bioleaching from lepidolite by *A. niger* fungus may be attributed to combination of biochemical (due to organic acids production) and biomechanical (due to hyphae penetration) leaching mechanisms. Significant drop of pH values indicates increased concentration of organic acids in the media as the result of high metabolic activity of the *A. niger* cell what was confirmed by various authors studying bioleaching by the microscopic fungi^14,22–25^. However, lepidolite interpenetration by *A. niger* hyphae growing along cleavages was observed by SEM analysis of solid residue after bioleaching, as well (Supplementary Information, Fig. S1), suggesting that direct biomechanical deterioration of lepidolite was also a part of the whole lithium extraction mechanism. However, according to Gadd^26^ the biochemical activities of microorganisms play more significant role than mechanical degradation.

Mechanisms of lepidolite bioleaching by bacteria is unknown. However, from abovementioned results it is obvious that no other substance except H^+^ ions contributed to the dissolution of Li^+^ ions. These results suggested that Li in lepidolite was dissolved by acid. Probably the mechanisms suggested by Liu et al.^20^ for leaching of lepidolite in sulphuric acid may be applied to bioleaching by acidophilic bacteria with sulphuric acid as a main bioleaching agent, as well. The main reaction of mixed alkali metal bioleaching may be expressed as follows:

1 $$ {\text{M}}_{{2}} {\text{O }} + {\text{ H}}_{{2}} {\text{SO}}_{{4}} = {\text{ M}}_{{2}} {\text{SO}}_{{4}} + {\text{ H}}_{{2}} {\text{O}} $$

where M presents alkali metals. Metallic elements from lepidolite are dissolved to form metal sulphates and mixed alums in the solution resulting just in partial lepidolite dissolution^20^. Overal reaction of lepidolite bioleaching in sulphuric acid produced by bacteria may be adopted from Onalbaeva et al.^11^:

2 $$ {\text{3Li}}_{{2}} {\text{O}}\cdot{\text{2K}}_{{2}} {\text{O}}\cdot{\text{5Al}}_{{2}} {\text{O}}_{{3}} \cdot{1}0{\text{SiO}}_{{2}} \cdot{\text{2SiF}}_{{4}} + { 2}0{\text{H}}_{{2}} {\text{SO}}_{{4}} = {\text{ 3Li}}_{{2}} {\text{SO}}_{{4}} + {\text{ 2K}}_{{2}} {\text{SO}}_{{4}} + {\text{ 5Al}}_{{2}} \left( {{\text{SO}}_{{4}} } \right)_{{3}} + {\text{ 11SiO}}_{{2}} + {\text{ H}}_{{2}} {\text{SiF}}_{{6}} + {\text{ 18H}}_{{2}} {\text{O }} + {\text{ 2HF}} $$

3 $$ {\text{3Li}}_{{2}} {\text{O}}\cdot{\text{2K}}_{{2}} {\text{O}}\cdot{\text{5Al}}_{{2}} {\text{O}}_{{3}} \cdot{\text{12SiO}}_{{2}} \cdot{\text{4H}}_{{2}} {\text{O }} + { 2}0{\text{H}}_{{2}} {\text{SO}}_{{4}} = {\text{ 3Li}}_{{2}} {\text{SO}}_{{4}} + {\text{ 2K}}_{{2}} {\text{SO}}_{{4}} + {\text{ 5Al}}_{{2}} \left( {{\text{SO}}_{{4}} } \right)_{{3}} + {\text{ 12SiO}}_{{2}} + {\text{ 24H}}_{{2}} {\text{O}} $$

Also Guo et al.^27^observed that increased H^+^ concentration catalysed the process of Li leaching from lepidolite via accelerating the protonation of the crystal lattices.

### X-ray diffraction analysis

XRD analysis was applied in this study for phase identification and structural changes evaluation of samples before and after bioleaching in all three studied systems. Significant differences in mineralogical composition of leaching residue among the three studied bioleaching systems are visible from XRD spectra comparison (Supplementary Information, Fig. S2) suggesting that different mechanisms can be responsible for bioleaching. While bacterial bioleaching led to the disappearing of muscovite phase from XRD spectrum, the fungal bioleaching led to the appearance of new silicate phase (SiO_2_) and muscovite was found a dominant phase. According to Liu et al.^20^ presence of quartz in the spectrum at the end of the process may correspond with alkali metal dissolution from the silicate lattice. Phase changes were observed also after bioleaching by yeast *R. mucilaginosa*. Reallocation and significant decrease of diffraction peaks intensity was observed and similarly as in case of microscopic fungi muscovite has become a dominant phase while polylithionite phase significantly weakened. Based on the results, it can be suggested that the bioleaching mechanisms of lepidolite by fungi and yeast may be similar, however, in the case of bacteria the mechanisms might be significantly different. Further experiments are necessary to understand the mechanisms behind the lepidolite bioleaching.

### Li distribution

**Figure 4:**
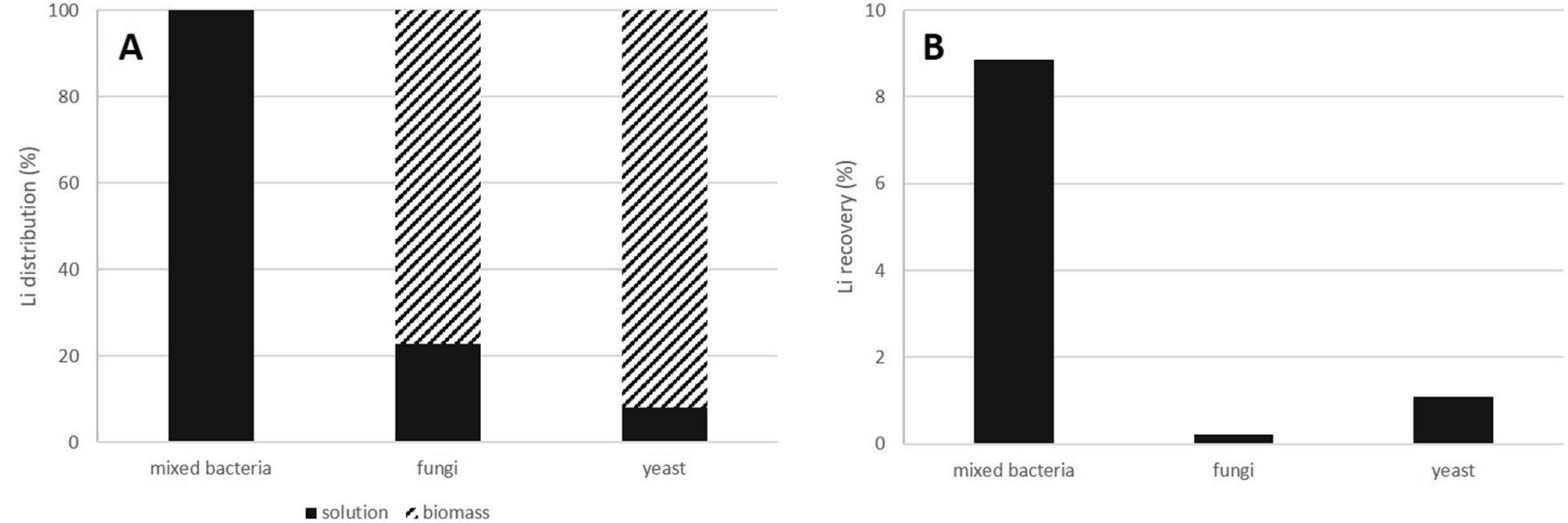
Distribution of Li between solution and biomass during bioleaching of lepidolite (**A**) and efficiency of the lepidolite bioleaching (**B**) by consortium of *A. ferrooxidans* and *A. thiooxidans* (bacteria), *A. niger* (fungi) and *R. mucilaginosa* (yeast) (fungi: initial ore concentration 10 g l^−1^, t = 21 °C, pH = 5.1, statically, standard medium, yeast: 10 g l^−1^, t = 21 °C, pH = 5.1, shaking 160 rpm, rich medium and bacteria: 10 g l^−1^, t = 30 °C, pH = 1.5, statically, poor medium).

Bioaccumulation of lithium into the biomass was observed when heterotrophic microorganisms *A. niger* and *R. mucilaginosa* were used (Fig. 4A). No bioaccumulation was found when bioleaching by consortium of acidophilic bacteria was studied. It can be suggested that the process of Li recovery by *A. niger* and *R. mucilaginosa* is a combination of two basic processes – initial bioleaching (metal solubilisation) followed by rapid bioaccumulation (intracellular lithium accumulation). It is possible that lithium bioaccumulation could significantly contribute to its solubilisation as released Li^+^ cations were fast accumulated in the cells and thus “pulled” the equilibrium resulting in the increased efficiency of the Li dissolution.

The highest amount of lithium was accumulated by *R. mucilaginosa* cells, representing 92% of the total amount of Li recovered from the ore. In the case of microscopic fungi *A. niger*, produced biomass accumulated 77% of the total solubilised Li. Distribution of Li between solution and biomass of particular microorganisms is shown in Fig. 4A. It is obvious that in both cases (fungi and yeast) bioaccumulation is dominant process of Li recovery and just small amount of Li^+^ ions remain in solution.

### Bioleaching efficiency

The bioleaching efficiency is given as a sum of two processes – Li dissolution and its accumulation in the biomass. The final bioleaching yields for consortium of *A. ferrooxidans* and *A. thiooxidans*, fungi *A. niger* and *R. mucilaginosa* were found to be 8.8%, 0.2% and 1.1%, respectively. The results suggested that the most efficient among all three studied systems was the consortium of acidophilic bacteria *A. ferrooxidans* and *A. thiooxidans* (Fig. 4B) with the final bioleaching yield of almost 9%. On the other hand, very long time (336 days) was necessary for the process. Reichel et al.^15^ found 11% Li recovery from zinnwaldite using consortium of sulphur-oxidising bacteria, however, authors reported just 14 days for observed Li bioleaching efficiency although they do not found clear explanation of higher bioleaching efficiency in comparison with chemical leaching.

The lowest bioleaching yield was observed when *A. niger* was used. Rezza et al.^13,14^ used *A. niger* for Li bioleaching from spodumene with highest recovery of 0.75 mg l^−1^ of lithium, they do not reported any bioaccumulation.

Composition of medium had very strong effect on bioleaching efficiency by *R. mucilaginosa* as in nutrient rich medium due to significantly higher biomass production majority of Li has accumulated into the biomass resulting in 3 times higher final Li recovery. There were also morphological differences observed between yeasts cultivated in nutrient rich and poor environments with spherical shape and thin exopolymer layer of 0.48 µm for yeast from nutrient rich media in comparison with oval cells and thick exopolymer layer (1.8 µm) when cultivated in nutrient poor medium^17^.

Despite of quite low bioleaching efficiency there is clearly visible potential of all three biological systems for Li recovery from hard rocks. Even with low Li concentration in solution after bioleaching, the lithium concentration in the leaching solution resembles the lithium concentration of sea water (0.1–0.2 mg l^−1^) and brines (0.1–2 g l^−1^) considered for economic recovery^28,29^. That shows that the leaching solution is generally suitable for further processing^15^.

Due to the expensive separation of Li from leaching liquor, the conventional processing routes are likely not economic. However, ability of fungus *A. niger* and especially yeast *R. mucilaginosa* represent advantageous route of Li recovery after bioleaching. Thermal, chemical or microbiological process can be used to Li extraction from the biomass later on.

Metabolic activity and hyphae penetration of microscopic fungi and yeasts resulted in significant structural changes of mineral enhancing the access of lithium by bioleaching agent. Maybe the combination of heterotrophic microorganisms (microscopic fungi or yeast) bioleaching leading to mineral structure changes with consequent bacterial bioleaching could bring better results in the future.

## Conclusions

The study describes the bioleaching of lithium from lepidolite using three different biological systems—acidophilic bacteria, microscopic fungus and yeasts. The results indicate that the presence of microorganisms was beneficial for Li bioleaching from lepidolite because no Li was found in abiotic controls. The lithium extraction was the highest using bacteria, however very long time was necessary for the process. The mechanisms of bioleaching by fungi and yeast differs from bacterial bioleaching. Significant deterioration of the mineral surface and structure were observed during fungal and yeast bioleaching after short time so probably the combination of two, heterotrophic followed by autotrophic processes would result in shortening of the time necessary for the process as well as increase of the bioleaching efficiency. Strong ability to accumulate Li represent the advantage of fungi and yeast exploitation for Li recovery from leachate. Thermal, chemical or microbiological process can be used to Li extraction from the biomass later on. The effect of medium composition was visible—to force bacteria to increase the rate and efficiency of bioleaching poor medium was suitable, however when bioaccumulation was the main aim, rich medium resulting in high biomass production was more advantageous.

**Table 1:** Mineralogical composition of the lepidolite (%).

SiO_2_	Al_2_O_3_	K_2_O	Li_2_O	Fe_2_O_3_	TiO_2_	CaO	MgO	Na_2_O
51	26.03	7.75	3.79	0.50	0.03	0.05	0.05	0.38

## Supplementary information

Supplementary information
