# Supplementary material for: Comparison of three different bioleaching systems for Li recovery from lepidolite
Source: Sci Rep. 2020 Sep 3;10:14594. doi: 10.1038/s41598-020-71596-5 (PMC7471267; doi:10.1038/s41598-020-71596-5)
Supplement: Supplementary file 1 — Supplementary information [file 41598_2020_71596_MOESM1_ESM.pdf]

Supplementary Information

Comparison of three different bioleaching systems for Li recovery from lepidolite

Sedlakova-Kadukova[1*], Jana; Marcincakova[2], Renata; Luptakova[3], Alena; Vojtko[4], Marek; Fujda[2], Martin; Pristas[1], Peter

[1]Faculty of Science, Pavol Jozef Safarik University in Kosice, Srobarova 2, 04154 Kosice, Slovakia, tel. +421-55-234-1228, jana.sedlakova@upjs.sk

[2]Faculty of Material, Metallurgy and Recycling, Technical University of Kosice, Letna 9, 04200 Kosice, Slovakia

[3]Institute of Geotechnics, Slovak Academy of Sciences, Watsonova 45, 04001 Kosice, Slovakia

[4]Institute of Materials Research, Slovak Academy of Sciences, Watsonova 47, 04001 Kosice, Slovakia

*corresponding author

Corresponding author address:

Department of Microbiology, Institute of Biology and Ecology, Faculty of Science, Pavol Jozef Safarik University, Srobarova 2, 04154 Kosice, Slovakia,

Tel: +421-55-2341228

Fax: +421-55-6222124

E-mail address: jana.sedlakova@upjs.sk

5 Pages, 2 Figures

Figure list:

Figure S1 Attachment of *A. niger* to lepidolite surface (A) and *A. niger* hyphae penetrating into the mineral (B)

Figure S2 XRD pattern of initial ore (A) and bioleaching residua after the bioleaching of lepidolite by consortium of *A. ferrooxidans* and *A. thiooxidans* (bacteria) (B), *A. niger* (fungi) (C) and *R. mucilaginosa* (yeast) (D)

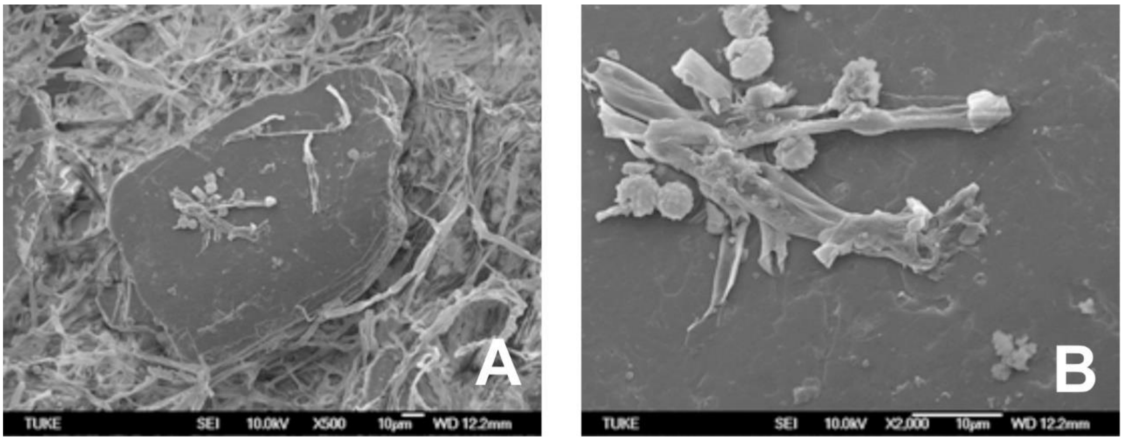

Figure S1 Attachment of *A. niger* to lepidolite surface (A) and *A. niger* hyphae penetrating into the mineral (B)

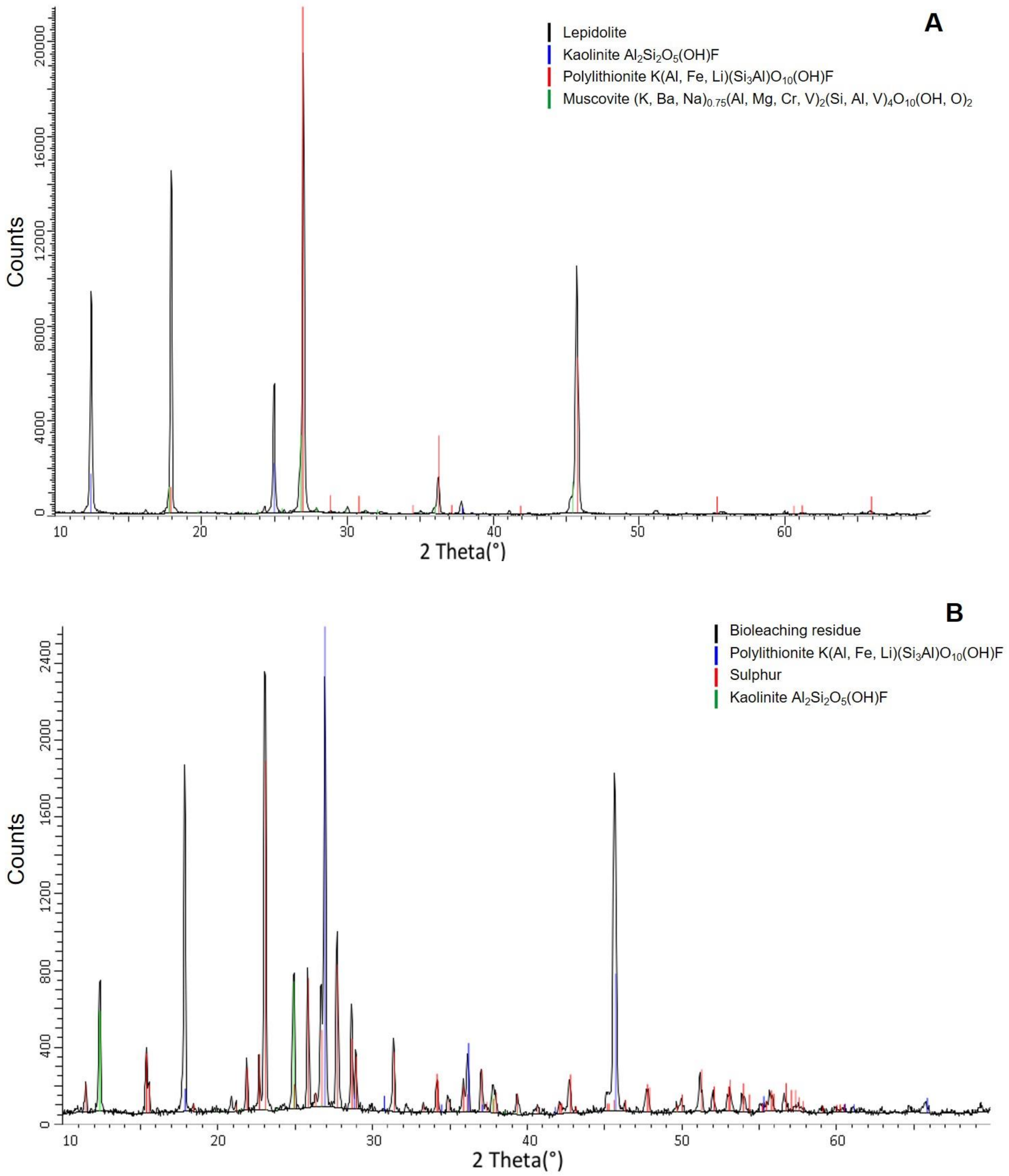

Figure S2 XRD pattern of initial ore (A) and bioleaching residua after the bioleaching of lepidolite by consortium of *A. ferrooxidans* and *A. thiooxidans* (bacteria) (B), *A. niger* (fungi) (C) and *R. mucilaginosa* (yeast) (D)

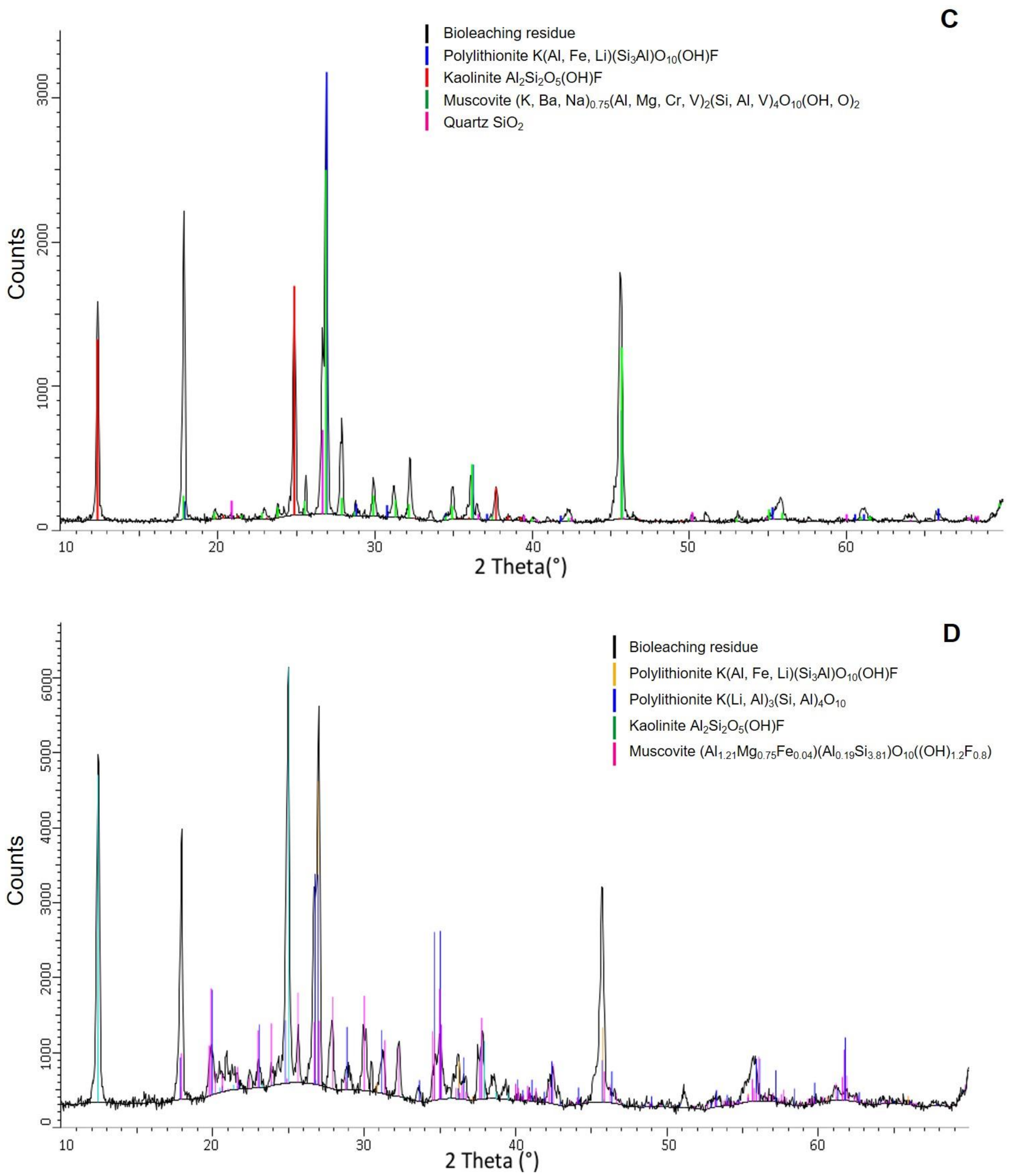

Figure S2 continued XRD pattern of initial ore (A) and bioleaching residua after the bioleaching of lepidolite by consortium of *A. ferrooxidans* and *A. thiooxidans* (bacteria) (B), *A. niger* (fungi) (C) and *R. mucilaginosa* (yeast) (D)
